# Introduction to the Special Issue “Understanding Brain Disease with Live Imaging”

**DOI:** 10.1117/1.NPh.12.S1.S14601

**Published:** 2025-08-29

**Authors:** Adam Q. Bauer, Nozomi Nishimura, Bistra Iordanova

**Affiliations:** a Washington University School of Medicine, St. Louis, Missouri, United States; b Cornell University, Meinig School of Biomedical Engineering, Ithaca, New York, United States; c University of Pittsburgh, Swanson School of Engineering, Pittsburgh, Pennsylvania, United States

## Abstract

The editorial introduced the Neurophotonics Special Issue Understanding Brain Disease with Live Imaging

Brain diseases arise from a broad range of causes including genetic factors, infection, acute/progressive injury, and lifestyle choices. From these conditions, alterations in the brain’s structure and/or function can ultimately lead to changes in behavior. Understanding the etiology and pathophysiology of different brain disorders therefore requires examination across different biological scales (cells to systems), over time, and ideally *in vivo*. Optical methods are uniquely suited to meet these challenges as current technology allows for longitudinal imaging across a wide range of spatiotemporal scales in awake behaving individuals. The present issue highlights a breadth of neurophotonic tools, including intravital imaging in animal models, to better understand disease onset and progression with the goal of reaching a broad scientific audience engaged in translational research into brain disease.

Vascular dysfunction is a critical contributor to Alzheimer’s disease (AD), and optical imaging has enabled mechanistic examination of this relationship in animal models. In this issue, multiple studies use live imaging to probe vascular and neuronal function in the context of AD.^1^^–^^5^ Eyre et al.^1^ investigate the correlation between cardiovascular risk factors and the incidence of AD noted in patients by layering atherosclerosis onto more traditional mouse models of Alzheimer’s that overexpress amyloid. In a separate study, Eyre et al.^2^ also characterize locomotion-induced hemodynamic responses across vascular compartments of the whisker barrel cortex of the same AD model. O’Connor et al.^3^ investigate arterial low-frequency oscillations in the frequency range associated with vasomotion in a preclinical model of AD to determine whether characterizing vasomotion could potentially act as a biomarker for the vascular dysfunction that accompanies AD. Crouzet et al.^4^ incorporate *in vivo* and *ex vivo* techniques to better understand cerebrovascular changes in a novel mouse model of late-onset, sporadic AD. In addition to the vascular contributions to AD, the decrease in synaptic density and neurodegeneration are still central to cognitive decline. *In vivo* optical approaches are well-suited to characterize real-time changes in axonal morphology during disease progression. Fulopova et al.^5^ explore the potential therapeutic benefits of transcranial stimulation to alter axonal morphology and improve synaptic plasticity in AD. Collectively, these valuable additions provide a platform for studying and understanding genetic, aging, and environmental factors driving AD pathology, potential biomarkers, and therapeutic approaches.

Some physiology, such as blood flow, can only be assessed in the whole living organism, and neurophotonics provides an excellent tool for linking blood circulation and disease across multiple scales.^6^^–^^8^ Chalard et al.^6^ combine intravital imaging with computational methods in a model of hemorrhagic stroke to explore how alterations in capillary network blood flow are detrimentally altered. Bowen et al.^7^ utilize wide-field optical imaging of calcium and hemodynamic activity in mice to demonstrate that early changes in spatiotemporal dynamics of remapped circuits and global networks predict functional recovery after stroke. In a model of vascular dysfunction, Bakker et al.^8^ observe regional changes in neurovascular coupling over the cortex, as well as changes in resting state network connectivity in mice with rigidified carotid arteries.

Going beyond vascular disease, Agner et al.^9^ evaluate cortical functional connectivity in an adult murine model of Zika virus infection using wide-field optical imaging of calcium and hemodynamic activity, in conjunction with extensive immunohistochemical analyses of neuron and synapse loss and myeloid and astrocyte activation. Niemeyer et al.^10^ use wide-field calcium imaging to map anatomical and functional networks and determine how their manipulation affects seizure propagation across the cortex in acute and chronic models of epilepsy. Du et al.^11^ review the development and applications of a multimodal imaging platform (leveraging light absorbance, fluorescence, and optical coherence tomography) to understand the effects of acute and chronic cocaine on the regulation of the neuro-astroglio-vascular unit. This thorough review demonstrates cocaine’s effects on vascular function and the underlying neural and glial changes that occur during cocaine administration.

Translational neuroscience benefited from the recent development of optical tools for measuring neural activity with calcium indicators, in part through large-scale efforts such as the US BRAIN Initiative.^12^ These capabilities to record from many neurons required advances in fast imaging, here comprehensively and insightfully reviewed by Nguyen et al.^13^ Recent advances in microscopy preparation include, for example, using a viscocohesive hyaluronan gel, which was demonstrated by Morton and Kim^14^ as an alternative immersion medium that is index-matched to water but dramatically enhances the stability of imaging for sustained time-lapse and physical arrangements previously unamenable to water. These collective advances can now be turned to the study of neural circuitry and cellular dysfunction in disease. This application is demonstrated by the valuable review on cortical population imaging by Silva et al.^15^ that surveys optical tools in the context of imaging internal state circuits relevant to psychiatric disease. The field also benefited from the development of new analytical tools for mesoscopic imaging of cortical neuronal and hemodynamic activity. A software package (umIT or “Universal Mesoscale Imaging Toolbox”),^16^ developed by the Vanni and Balbi labs, provides an accessible, user-friendly interface for examining wide-field optical imaging data amenable to trainees and experienced users alike.

Early developments in optical live imaging were focused primarily on basic neuroscience to understand how the brain works. The present issue highlights opportunities for wider application of neurophotonics beyond the scope of basic science and into translational research in brain disease. Across many brain diseases, treatment options are limited in number and efficacy; we are thus in dire need of engaging and supporting research that spans and integrates across basic and translational levels. Purely clinical approaches do not allow for single-cell and circuit *in vivo* dissection of disease pathways, capabilities provided by methods outlined in this issue. In the evolving search for brain therapies, we believe that live optical imaging has an important role in linking basic and clinical approaches, and we advocate for making neurophotonics more accessible for translational researchers and clinician-scientists.
